# Spatial serosurvey of anti-*Toxoplasma gondii* antibodies in individuals with animal hoarding disorder and their dogs in Southern Brazil

**DOI:** 10.1371/journal.pone.0233305

**Published:** 2020-05-15

**Authors:** Graziela Ribeiro da Cunha, Maysa Pellizzaro, Camila Marinelli Martins, Suzana Maria Rocha, Ana Carolina Yamakawa, Evelyn Cristine da Silva, Andrea Pires dos Santos, Vivien Midori Morikawa, Hélio Langoni, Alexander Welker Biondo

**Affiliations:** 1 Department of Veterinary Medicine, Federal University of Paraná State, Curitiba, Paraná, Brazil; 2 Institute of Collective Health, Federal University of Bahia, Salvador, Bahia, Brazil; 3 Department of Nursing and Public Health, Ponta Grossa State University, Ponta Grossa, Paraná, Brazil; 4 AAC&T Research Consulting, Curitiba, Paraná, Brazil; 5 Department of Veterinary Hygiene and Public Health, School of Veterinary Medicine and Animals Science, São Paulo State University, Botucatu, São Paulo, Brazil; 6 Department of Comparative Pathobiology, College of Veterinary Medicine, Purdue University, West Lafayette, Indiana, United States of America; 7 Department of Collective Health, Federal University of Paraná State, Curitiba, Paraná, Brazil; 8 Secretary of Environment, Curitiba City Hall, Curitiba, Paraná, Brazil; Faculty of Science, Ain Shams University (ASU), EGYPT

## Abstract

Despite vulnerability and unsanitary conditions of animal hoarding may predispose environmental contamination and spread of vectors and pathogens, no study to date has focused on their impact on public health and zoonotic diseases. Accordingly, this study aimed to assess the seroprevalence of anti-*Toxoplasma gondii* antibodies and associated factors in individuals with animal hoarding disorder (AHD) and their dogs in Curitiba, Southern Brazil. Blood samples were obtained from 264 dogs (21 households) and 19 individuals with AHD (11 households). Their blood was tested by indirect fluorescent antibody test (IFAT). Overall, anti-*Toxoplasma gondii* seropositivity was found in 21/264 dogs (7.95%; 95% CI: 4.69–11.22) with titers ranging from 16 to 4096, and in 7/19 individuals with AHD (36.84%; CI: 15.15–58.53) with titers ranging from 16 to 64. Serological analysis for anti-*T*. *gondii* antibodies were considered positive in at least one individual or dog in 9/11 (81.82%; 95% CI: 59.03–100.00) cases that were thoroughly assessed. Surprisingly, the seropositivity of individuals with AHD and their dogs was among the lowest reportedly observed in human and dog populations of Brazil. There was no significant association between positive owners and positive dogs or the presence of cats in the household. Regard epidemiological variables, a significant association was found between dog’s seropositivity and the type of dog food. To the authors’ knowledge, the present study represents the first investigation of *T*. *gondii* seroprevalence in individuals with hoarding disorder and their dogs. In conclusion, despite low sanitary conditions, anti-*Toxoplasma gondii* antibodies frequency in individuals with AHD and their dogs are lower than the general population likely due to low protozoan load in such isolated households.

## Introduction

Hoarding disorder has been considered a serious threat to public health, mainly due to frequent unsanitary household conditions, which may favor pathogen amplification and disease spreading [1,2], posing as health risks to the individuals themselves, their companion animals, and surrounding neighborhoods [3,4]. Despite animal hoarding, a particular manifestation of hoarding disorder [5] has been early described as “owners of many pets” [6]; the definition refers more to the lack of providing minimum personal and animal care than the number of owned animals at risk [7].

The quality of life of people with hoarding disorder can be considerably impaired because of precarious conditions, which may predispose to numerous health hazards [8,9] and create a vulnerable situation for both the individuals with hoarding behavior and their companion animals. Animal involvement may worse the unhealthy living conditions, mainly due to their poor perception leading to failure in keeping adequate animal and environmental care [5,8]. Animals in hoarding situations reportedly lack proper food, water, veterinary care, and live in crowded and unsanitary spaces with feces and urine accumulation [7]. Although several clinical conditions and injuries may be commonly observed in hoarded animals [10–13], few studies have specifically addressed the problem [14–16]. Although the risk of zoonotic disease transmission has been a particular concern in animal hoarding situations [2,12], only a single study has focused on potential zoonotic nematodes in cats under hoarding situations [15].

*Toxoplasma gondii* has been described as an intracellular parasite infecting all warm-blooded animals and causing a worldwide spread zoonosis called toxoplasmosis [17]. Toxoplasmosis has been reportedly considered one of the most common foodborne parasitic infection, acquired by ingestion of contaminated water, food, raw or undercook meat [18]. The human disease has been usually asymptomatic, but clinical manifestations are concerning to immunosuppressed individuals and pregnant women, which may lead to fetal injuries and abortion [17,19]. Increased risk of human toxoplasmosis infection may also associated with socioeconomic and cultural factors, low income, and low educational levels [19–21].

Human T. *gondii* seroprevalence has reportedly ranged from 0.8% to 77.5% worldwide [17]. Studies from Latin American countries demonstrated significantly higher seropositivity rates [17], particularly in pregnant women. In Brazil, frequencies of anti-*T*. *gondii* antibodies in humans vary among different states and regions: northern region with 56.7% (131/231) in Amazonas [22], 65.8% (225/342) in Acre [23], northeastern region with 66.2% (1020/1540) in Rio Grande do Norte [24], central-western region with 97.4% (113/116) in Mato Grosso [25], southeastern region with 32.4% (110/339) [26] and 63.7% (618/970) [27] in São Paulo, and 36.0% (552/1532) in Minas Gerais [28], and southern region with 53.2% (183/344) in Rio Grande do Sul [29]. Studies performed at Paraná State have reported frequencies of 41.54% (248/597) in Londrina city [21], 73.57% (526/715) in Ivaiporã city [20], and 62.5% (50/80) among tissue donors from the state capital, Curitiba city [30] (S1 Table).

Although dogs may present a high likelihood of *T*. *gondii* infection due to their carnivorous behavior, their role on the parasite cycle is considered secondary, with rare clinical manifestations [31]. As observed in humans, the seroprevalence of anti-*T*. *gondii* in dogs have widely varied in Brazil, ranging from 9.54% (23/241) in Pernambuco [32] to 88.5% (54/61) in Mato Grosso [25]. Studies performed at Paraná State showed differences in frequencies of *T*. *gondii* antibodies in dogs: 16.32% (119/729) [21] and 20.6% (56/271) [33] in Londrina, 30.7% (8/26) in Curitiba [34], 67.02% (435/649) in Foz do Iguaçu [35] and 70.85% (124/175) in Umuarama [36] (S1 Table).

Despite the close and promiscuous human-dog contact, poor sanitary conditions, and environmental exposure, all of which favor human *T*. *gondii* infection [37], no study to date has focused on dogs in hoarding conditions as environmental sentinels of the human correspondent disease. Accordingly, this study aimed to assess the seroprevalence of *T*. *gondii* antibodies and associated factors in individuals with animal hoarding disorder (AHD) and their dogs in a metropolitan city of Southern Brazil.

## Materials and methods

### Study area

The study was conducted in a metropolitan city of Southern Brazil, Curitiba (25°25’47” S and 49°16’19” W), capital of Paraná State, Southern Brazil. This city has been ranked as the eighth biggest Brazilian city with approximately 1.9 million inhabitants [38]. Totally comprised by urban area and the coldest Brazilian state capital with annual average temperature of 16.5°C and a subtropical highland climate, Curitiba has been considered the best Brazilian city in sustainability and quality of life, with a very high human development index (HDI) of 0.823, ranked fourth among 26 state capitals and tenth among all 5,570 Brazilians cities [39].

### Sample collection

A recent study in Curitiba reported at least 65 confirmed animal hoarding cases, keeping a total of 724 dogs in 40 cases where in-household access has been allowed [40]. Based on this previous study, a minimum sampling of 251 dogs was reached in a simple random sample calculation designed with a 95% confidence level and 5% accuracy. Thus, all previously identified cases were visited during a one-year period to collect blood samples from dogs. Due to a delay on ethics committee and city’s secretary of health authorization, there was a second round of visits at same households to collect people sampling. In each household case, the convenience of as many as possible dog samplings was applied, along with all volunteered people living in the household.

House-to-house visits for dog samplings were carried out in accordance and along with the city’s secretary of environment and visits for people sampling along with the city’s secretary of health from 2017 to 2019. Canine blood samples were drawn by certified veterinarians, and certified nurses collected blood from people. Voluntarily signed consent was obtained before sampling, and procedures were performed in accordance with the National Brazilian Platform for the use of human data and samples. Because of exposure to the same hoarding conditions, all people and dogs living in the household case were considered as individuals with animal hoarding disorder (AHD) and hoarding dogs for statistical purposes, regardless of the exposed period prior to sampling.

### Epidemiological data collection

Epidemiological data were obtained by applying a questionnaire with objective questions regarding AHD individuals and dog exposure to *T*. *gondii*. Data regarding perception about the infection and environment observation were also collected and analyzed. The questionnaire has been developed by the research group. The questions were based on individual self-report and visual household environment inspection and answered at the same visit performed for dog’s blood sample collection (S1 Questionnaire).

Variables used to investigate AHD individual exposure included the use of gloves to collect the animals' feces, habit of eating raw or undercooked meat, previously reference of hearing about toxoplasmosis, cat hoarding, object hoarding, and presence of cats in the household. Regarding dog exposure, investigated variables included living place, food type, and feeding place (S1 Questionnaire).

Regarding to the household conditions, the investigated variables included the presence of cats, vegetable garden, open sewer or stream near the house, cat hoarding, object hoarding, feces on floor, remains of food, and trash in the yard. Besides, the situation of food preparation place, house features, and backyard features were investigated (S1 Questionnaire).

### Serological diagnosis

Serum samples were tested for specific IgG antibodies against *T*. *gondii* by the indirect fluorescent antibody test (IFAT) [41]. Serial dilutions of 1:16, 1:64, 1:256, 1:1024, and 1:4096 were performed in pH 7.2 phosphate-buffered saline solution (PBS: 8.2g NaCl, 1.9g Na_2_HPO_4_7H_2_O and 0.3g NaH_2_PO_4_H_2_O per liter). Immunofluorescence slides were previously sensitized with 0.1% formaldehyde to inactivated tachyzoites of *T*. *gondii* (RH strain). For the fluorescence, a commercial anti-IgG antibody specific to humans or dogs, conjugated with the fluorescein isothiocyanate (Bethyl—Montgomery, TX, USA), was used for the respective species. Samples were considered positive if antibody titers were ≥ 16 for *T*. *gondii* as the established cut-off, and final titers were determined to the last dilution at which ≥ 50% of the tachyzoites presented fluorescence.

### Statistical analysis

The epidemiological data and frequencies were stratified in three dependent variables to develop statistical analysis: (1) households fully assessed (i.e., cases in which people and dogs were sampled), (2) dogs sampled, and (3) AHD individuals sampled. For each dependent variable, independent variables were selected from the epidemiological questionnaire to evaluate association with *T*. *gondii* seropositivity.

All variables were evaluated using descriptive and bivariate analyses with frequencies (simple and cross-tables), estimation of ORs (confidence intervals of 95%) and chi-square test (significance level = 0.05) provided by a commercial statistical software (SPSS for Windows, version 16.0, SPSS Incorporated, Chicago, IL, USA). Pearson’s coefficient was used to evaluate the correlation between the number of positive people and the number of positive dogs at the same house.

Figures were produced with the distribution of collected data. Corresponding geographic coordinates were obtained for each case sampled, and a free Brazilian geodatabasis [42] was used in ArcGIS 10 [43]. A kernel density map was generated for the evaluation of *T*. *gondii* seropositivity in dogs and households with positive individuals with AHD’s distribution using the density.ppp function of the “spatsat” package [44] in the R environment [45]. Thus, all data sources used in the analysis were owned by the authors or freely accessible.

### Ethics statement

This study was approved by the National Human Ethics Research Committee (protocol number 3,166,749/2019) and the Animal Use Ethics Committee (protocol number 077/2015), both through the Federal University of Paraná, Southern Brazil.

## Results

Dog sampling was allowed in 21 households, totalizing 264 dog samples (out of a total of 550 dogs). Likewise, in 11 households, people allowed their blood collection, totalizing 19 people samples.

Seropositivity for *T*. *gondii* antibodies was observed in 21/264 (7.95%; 95% CI: 4.69–11.22) dogs, with titers ranging from 16 to 4096. The proportion of seropositive dogs per case ranged from 6.25% to 53.85%, whereas 7/19 (36.84%; CI: 15.15–58.53) AHD individuals were positive, with titers ranging from 16 to 64. Considering the cases in which dogs and people were sampled, serological analyses were positive in at least one of them (individual with AHD or dog) in 9/11 (81.82%; 95% CI: 59.03–100.00) households. Simultaneous seropositivity of people and dogs was found in 3/11 (27.27%) cases, only positive people samples in 3/11 (27.27%) cases, and only positive dog samples in 3/11 cases (27.27%).

No significant association was found between the presence of seropositive people and the presence of seropositive dogs (OR 0.6, p = 0.60) in the eleven fully assessed households. Households with seropositive people presented an average of 12.87% seropositive dogs. No significant correlation was found between the number of positive people and the proportion of positive dogs per case (r = 0.105; p = 0.75). Regarding the 19 sampled people, no significant association was found between positive dogs and AHD individuals (OR 0.9; p = 0.66).

The two epidemiological variables associated with seropositive dogs were dogs’ food type (p <0.01), and people’s report knowledge about toxoplasmosis (p = 0.01) (Table 1). No significant association was observed in the bivariate analysis between the investigated epidemiological variables and seropositivity in AHD individuals and fully assessed households (S2 Table).

**Table 1:** Bivariate analysis of epidemiological data and seropositivity for anti-*T*. *gondii* in dogs from individuals with animal hoarding disorder (AHD) in Curitiba, Paraná, Brazil.

Dogs (N = 264)		Positive n (%)	Total	OR	95% CI	p-value
Living place	Inside home	0 (0.0)	4			*
	Backyard	19 (8.7)	218	-	-	*
	Both	2 (4.8)	42	-	-	*
Food type	Commercial dog food and home-cooked food	14 (5.9)	239	0.1	0.05–0.44	<0.01
	Commercial dog food	7 (28.0)	25			
Feeding place	Food bowls	14 (8.5)	164	1.2	0.48–3.18	0.65
	On the floor	7 (7.0)	100			
Knowledge about toxoplasmosis	Yes	9 (4.7)	191	0.2	0.11–0.74	0.01
	No	9 (15.0)	60			
Cat hoarding	Yes	8 (6.6)	121	0.7	0.28–1.77	0.30
	No	13 (9.1)	143			
Object hoarding	Yes	6 (7.1)	85	0.8	0.31–2.22	0.45
	No	15 (8.4)	179			
Presence of cats in the household	Yes	16 (8.2)	194	1.1	0.41–3.32	0.50
	No	5 (7.1)	70			
Remains of food	Yes	7 (7.5)	93	0.8	0.33–2.21	0.47
	No	14 (8.6)	162			

* There was no sufficient exposed and no exposed to proceed the analysis.

**Fig 1:**
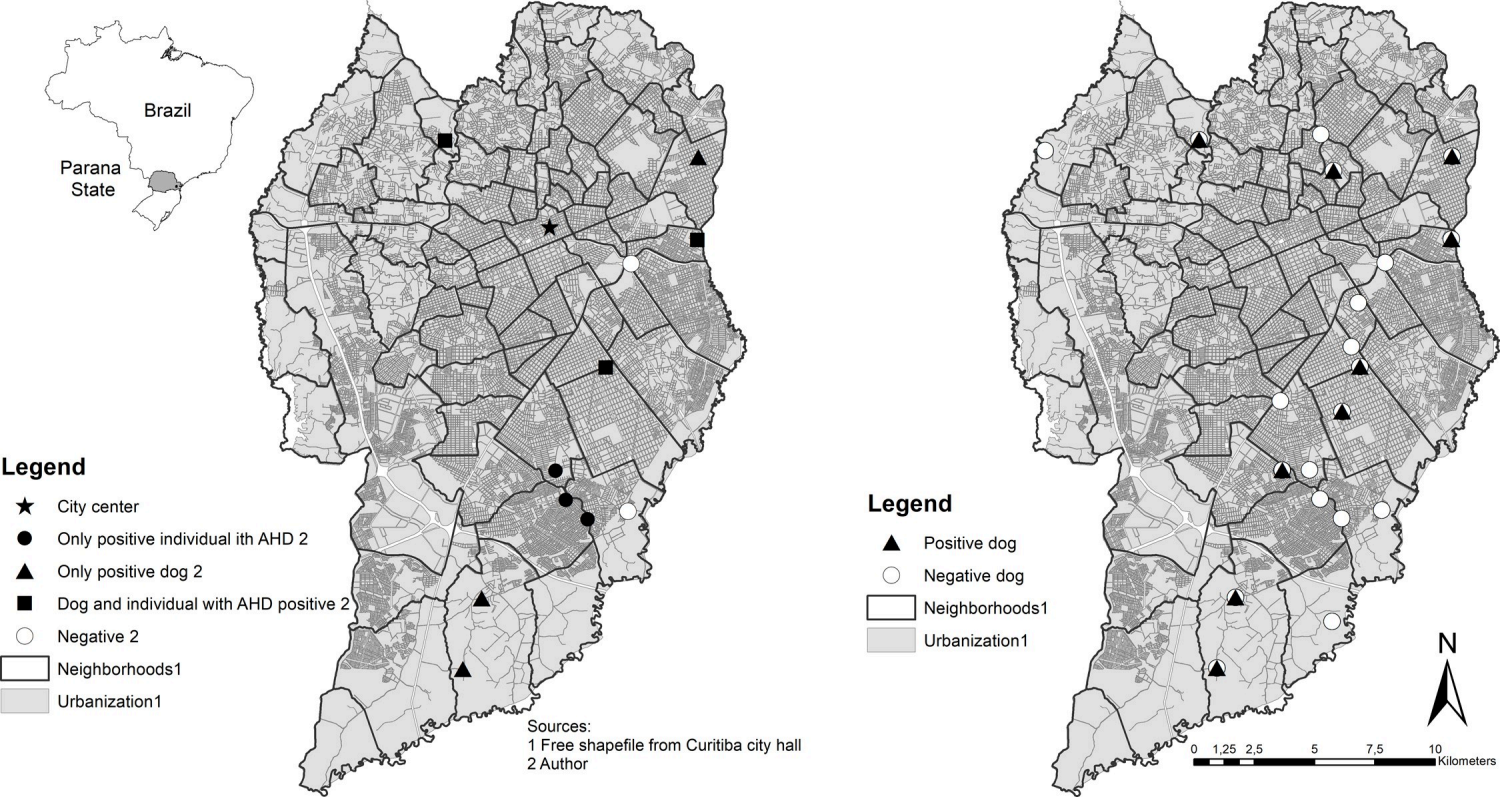
Spatial distribution per serological *T*. *gondii* status of 11 thoroughly assessed households (left) and 21 households where hoarded dogs were sampled (right) in Curitiba, Paraná, Brazil.

**Fig 2:**
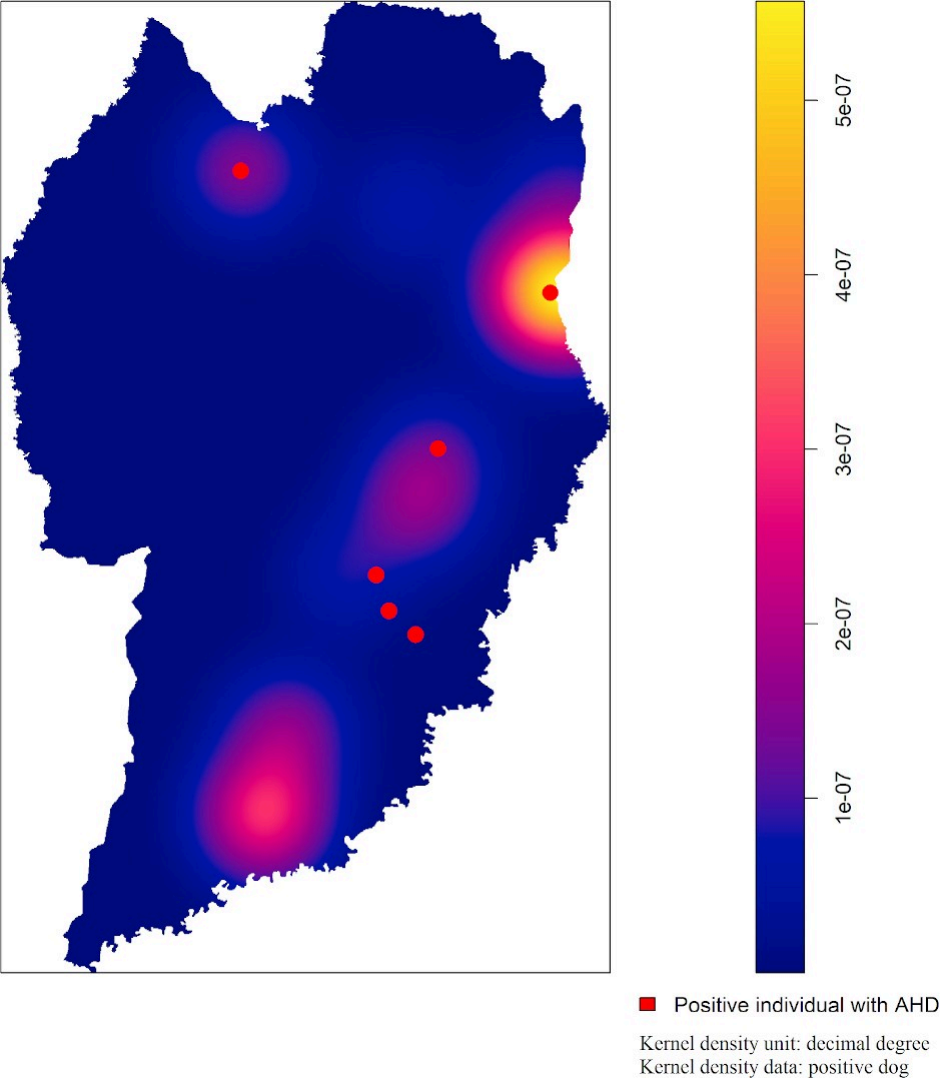
Kernel density analyses of positive dogs to anti-*T*. *gondii* antibodies (colored from yellow in hot areas to blue in cold areas) with positive individuals with animal hoarding disorder (AHD) in overlapping points (red) in Curitiba, Paraná, Brazil. The map has been produced by authors, using free open access shapefiles described in methodology section.

Geographical distribution was gathered for the eleven fully assessed households and all 21 households where dogs were sampled and presented according to seropositivity to *T*. *gondii* (Fig 1). A heat area in the Kernel map has shown the occurrence of a higher intensity number of seropositive dogs in one case in the east zone of the city, in the same area where a positive AHD individual was identified (Fig 2).

In the right map, as two cases were very close together, only one point can be seen in this scale. The map has been produced by authors, using free open access shapefiles described in methodology section.

## Discussion

To the authors’ knowledge, the present study represents the first investigation of *T*. *gondii* seroprevalence in individuals with animal hoarding disorder (AHD) and their dogs. Few studies conducted in Brazil have previously investigated *T*. *gondii* seroprevalence simultaneously between people and their dogs [21,25]. Surprisingly, the seropositivity of ADH individuals and their dogs herein were among the lowest reportedly observed in human and dog populations of Brazil (S1 Table).

The results herein have shown that frequency of households with simultaneous seropositivity of *T*. *gondii* between people and their dogs (27.27%) was the same as in households with only-positive dogs and only-positive ADH individuals (27.27%) (Fig 1), suggesting that the presence of dogs may have no impact for serological *T*. *gondii* status in individuals with hoarding behavior. Despite differences in frequencies, such results have corroborated with a previous study in which the intra-domicile environment had no impact on seropositivity for serological *T*. *gondii* status between owners and their dogs [21]. Although sharing environment between dogs and people may offer favorable conditions for infection [35], particularly when sharing potentially contaminated food and water [34], differences in frequencies in AHD individuals and their dogs found herein have rejected such assumptions. Out of studied cases, only one has shown high frequency of seropositivity in owned dogs and in ADH individuals in the same household (Fig 2).

As already established, sanitary conditions in both object and animal accumulation behavior may worse the risk of transmission of *T*. *gondii*. An excess of trash and dirt (feces, urine, secretions, food remnants) may predispose sporulation and spread of viable oocysts putting in risk the people and animals of the household [21]. Unexpectedly, no significant association was observed between *T*. *gondii* seroprevalence and all variables related to unsanitary conditions analyzed herein (object hoarding, open sewer, presence of feces and remains of food on floor, conditions of food preparation place, house and backyard features, and trash in the yard) (S2 Table).

Despite a previous report in Northeastern Brazil showing a higher chance of infection in individuals residing in dirt houses compared to houses with pavemented yards [24], another study in Southern Brazil has determined that risk factors such as the living area (urban or rural), yard hygiene, and contact with sand or land were not associated to seropositivity [20]. The low frequency found in the study herein has suggested a low protozoan load at the households or lack of ideal specific conditions [46].

Although cats have been the primary definitive host for *T*. *gondii* and source of environmental contamination in urban areas [47] shedding millions of environmentally resistant oocysts in their feces [48], the association between the presence of cats and seropositivity for *T*. *gondii* in humans and dogs remains controversial. Some studies have shown a significant association between the presence of cats and the risk of infection in human beings [24] and dogs [49]. In contrast, other studies have not confirmed such human [20,25,27] and dog [25,50] association. The presence of cats reported in most households (8/11; 72.72%) is similar to previously reported studies in Brazil [27,25]. The results presented herein show no association between the presence of cats and the seropositivity for *T*. *gondii* when considering households and AHD individuals groups. Also, no association between cat hoarding and seropositivity for *T*. *gondii* was found when considering individuals with AHD and households groups (S2 Table).

A study has shown that responsible ownership of domestic cats is associated with low toxoplasmosis seroprevalence, probably due to adequate food habits and restricted outdoors access [51], which also contributes to low local environmental contamination. These previous findings may explain the low protozoan load in the surveyed households of the present study, reinforcing the existence of many influential factors for *T*. *gondii* infection [46], beyond the presence of cats and unsanitary environmental conditions.

The frequency of *T*. *gondii* antibodies in AHD individuals found herein (36.84%) was amongst the lowest compared to the general human population range in Brazil (32.4% [26] to 97.4% [25]) (S1 Table), and significantly higher than the frequency found in their dogs (7.95%). The low number of human samples analyzed may have contributed to the low frequency. Similar discrepancies between healthy populations of people and dogs have been previously reported in other Brazilian cities [21,25], likely due to different food exposure as toxoplasmosis has been primarily considered a foodborne disease [18,21].

Although previous studies have shown that clutter and unsanitary conditions frequently found in AHD individual households may result in impairments of normal activities through daily living, such as preparing food [8,52,53], no study to date has shown zoonotic impact in hoarding cases. As observed, people with AHD commonly feed themselves with precooked, ready, and fast food or leftovers (usually provided from community donations) instead of fresh salad or fresh meat, which may have decreased the risk of *T*. *gondii* contamination. Moreover, no significant association was found between the seropositivity for *T*. *gondii* and their habit of eating raw or undercooked meat (S2 Table), probably due to a pre-frozen meat origin, which may kill viable *Toxoplasma*. Interestingly, a significant association was found between the knowledge about the disease to the low seropositivity of *T*. *gondii* in dogs, but not AHD individuals’ seropositivity.

Surprisingly, the frequency of anti-*T*. *gondii* antibodies in hoarded dogs herein (7.95%) was lower than the frequency found in dogs performed in nearby areas in general population [21,33–36] (S1 Table). Since environment with higher affective contact between dogs and tutors may provide favorable conditions for *T*. *gondii* infection [35], besides a potential low pathogen load in the household, the relatively lower affection observed between individuals with AHD and their own dogs may explain the lower serological frequency observed herein. Such low frequency may also be the consequence of the type of food provided to dogs.

A previous study conducted in indigenous communities demonstrated a significantly higher prevalence of *T*. *gondii* in dogs with hunting activity [54], while in another study, eating hunted meat was not statistically associated with seropositivity for *T*. *gondii* in dogs [50]. Despite low, the frequency of *T*. *gondii* seropositivity herein was significantly higher in dogs fed only with commercial dog food (p <0.01) (Table 1), contrasting to a previous study showing no significant association with the type of dog diet [49]. This association may suggest potential contamination of low-quality commercial dog food, or during household storage, considering the poor sanitary conditions, which were not explored herein and should be further investigated.

The variation in *T*. *gondii* frequencies reported in dogs of Brazil (9.54% [32] to 88.5% [25]) may be related to behavioral, socioeconomic, cultural, climate, and sanitary differences among Brazilian regions [55]. As previously indicated, dogs may serve as environmental indicators of pathogen circulation [34,35] and appropriate ecological conditions for parasite maintenance in dogs and human infections [36,56]. Finally, the frequency variation of serological *T*. *gondii* status in individuals with AHD and their dogs emphasizes the importance of the One Health approach to better understand the epidemiology and ecology of a pathogen [47] where environment, animal, and human aspects, particularly in vulnerable populations, should be considered.

As limitations, the present study has used a relatively low number of human samples and consequently hoarder locations, which may have generated insufficient data to represent all AHD individuals and their dogs. However, low sampling herein has been consequence of reportedly lack of social empathy and refusal by AHD individuals, with no other similar study to date. Additionally, the use of self-report information may have been influenced by biased response and incorrectly recall due to AHD and other possible concomitant disorders. Furthermore, as consequence of ethics committee and permission of city secretary of health, sampling dates were different in individuals with AHD and their dogs, which may have affected the results due to possible changes in *T*. *gondii* exposure overtime. Nevertheless, serological diagnosis herein has been on purpose based on IgG antibodies, which may have a lifetime duration. Despite such limitations, this study has been shown original and important findings on AHD population, hoarded dogs and their impact on public health. Likewise, the study has highlighted the lack of previous reports involving both individuals with AHD and their dogs, mainly due to difficulties in accessing households in developing countries, and further studies should be conducted to fully establish the sanitary impact of AHD individuals and their pets.

## Conclusion

Despite low sanitary conditions, the frequency of anti-*Toxoplasma gondii* antibodies in individuals with animal hoarding disorder and their dogs was lower than the general population, probably due to low protozoan load in the surveyed households. Despite usually unhealthy, food habits and isolation commonly observed in individuals with animal hoarding disorder may be protective factors associated with human exposure to *Toxoplasma gondii*.

## Supporting information

S1 Table Review of Brazilian studies investigating human and/or dog seropositivity for anti-*T*. *gondii* in different target populations and locations from 2001 to 2019. (PDF)

S2 Table Bivariate analysis of epidemiological data and seropositivity for anti-*T*. *gondii* in households and individuals with animal hoarding disorder (AHD) in Curitiba, Paraná, Brazil. (DOCX)

S1 Questionnaire Epidemiological questionnaire used to investigation of seroprevalence of anti-*T*. *gondii* antibodies and associated factors in individuals with animal hoarding disorder and their dogs in Curitiba. (PDF)
