# Supplementary material for: Spatial serosurvey of anti-Toxoplasma gondii antibodies in individuals with animal hoarding disorder and their dogs in Southern Brazil
Source: PLoS One. 2020 May 15;15(5):e0233305. doi: 10.1371/journal.pone.0233305 (PMC7228105; doi:10.1371/journal.pone.0233305)
Supplement: S1 Table — (PDF) [file pone.0233305.s001.pdf]

Supporting information

S1 Table: Review of Brazilian studies investigating human and/or dog seropositivity for anti-*T. gondii* in different target populations and locations from 2001 to 2019.

City/State	Human Target population	Human Seroprevalence Pos/Tot. (%)	Dog Target population	Dog Seroprevalence Pos/Tot. (%)	Reference
Jauru microregion/MT	Rural workers living in farms	113/116 (97.4)	Domiciled (living in farms)	54/61 (88.5)	(Santos et al., 2009)
Londrina/PR	Owners	248/597 (41.54)	Domiciled	119/729 (16.32)	(Benitez et al., 2017)
West area of São Paulo municipality/SP	Children	110/339 (32.4)	-	-	(Francisco et al., 2006)
Granada/AC (rural Amazonia)	Individuals between 5-90 years old	225/342 (65.8)	-	-	(Ferreira et al., 2009)
Ituiutaba/MG	Patients from a clinical analysis laboratory	552/1532 (36.0)	-	-	(Maia et al., 2012)
Lábrea/AM	Riverside communities	131/231 (56.7)	-	-	(Vitaliano et al., 2015)
Cassia dos Coqueiros/SP	Adults residents	618/970 (63.7)	-	-	(Passos et al., 2018)
Santa Cruz/RN	Adults residents	1020/1540 (66.2)	-	-	(Aloise et al., 2018)
Pelotas/RS	Rural population	183/344 (53.2)	-	-	(Araújo et al., 2018)
Curitiba/PR	Tissue donors	50/80 (62.8)	-	-	(Tuon et al., 2019)
Ivaiporã/PR	Citizen	526/715 (73.57)	-	-	(Mareze et al., 2019)
Northeast of São Paulo State /SP	-	-	Domiciled	151/295 (51.19)	(Varandas et al., 2001)
São Paulo/SP	-	-	Stray	101/200 (50.5)	(Meireles et al., 2004)
Salvador/BA	-	-	Stray	143/225 (63.55)	(Barbosa et al., 2006)
Ubatuba/SP	-	-	Domiciled	52/205 (25.4)	(Silva et al., 2010)
Umuarama/PR	-	-	Shelter dogs	124/175 (70.85)	(de Paula Dreer et al., 2013)

South of the Brazilian Amazon	-	-	Dogs living in Brazilian indigenous communities	169/325 (52.0)	(Minervino et al., 2013)
Ilha Solteira/SP	-	-	Dogs from rural area	44/93 (47.3)	(Paulan et al., 2013)
Londrina/PR	-	-	Birth Control Project of UEL	56/271 (20.6)	(Caldart et al., 2015)
Tocantins/TO	-	-	Domiciled	117/204 (57.4)	(Raimundo et al., 2015)
Pantanal and Amazon biomes of Brazil.	-	-	Domestic	49/128 (38.28)	(Furtado et al., 2015)
Pirassunungua/SP	-	-	Kennel and the city’s veterinary clinics	135/300 (45.0)	(Seabra et al., 2015)
Curitiba/PR	-	-	Neighborhood dogs	8/26 (30.7)	(Constantino et al., 2016)
Rio Cuiaba/MT	-	-	Dogs living in riverside communities	107/248 (43.1)	(Rodrigues et al., 2016)
Fernando de Noronha/PE	-	-	Domiciled	156/320 (48.75)	(Magalhães et al., 2017)
João Pessoa/PB	-	-	Dogs attended at veterinary clinics	37/384 (9.6)	(Brasil et al., 2018)
Garanhuns/PE	-	-	Domiciled	23/241 (9.54)	(Souza et al., 2019)
Foz do Iguaçu/PR	-	-	Domiciled	435/649 (67.02)	(Pinto-Ferreira et al., 2019)
Belo Horizonte/MG	-	-	Stray	31/93 (33.3)	(Saldanha-Elias et al., 2019)

References

Aloise DA, Coura-Vital W, Carneiro M, Rodrigues MV, Toscano GA da S, Silva RB da, et al. Seroprevalence and Risk Factors for Human Toxoplasmosis in Northeastern Brazil. Rev Patol Trop 2018;46:307.

Araújo AC, Villela MM, Sena-Lopes Â, Farias NA da R, de Faria LMJ, Avila LF da C, et al. Seroprevalence of Toxoplasma gondii and Toxocara canis in a human rural population of southern Rio Grande do Sul. Rev Inst Med Trop Sao Paulo 2018;60:1–7. doi:10.1590/s1678-9946201860028.

Barbosa MVF, Guimarães JE, Almeida MÂO, Gondim LFP, Regis GB. Freqüência de anticorpos IgG anti-Toxoplasma gondii em soros de cães errantes da cidade de Salvador-Bahia, Brasil. Brazilian J Vet Res Anim Sci 2006;40:457–65. doi:10.1590/s1413-95962003000600010.

Benitez A do N, Martins FDC, Mareze M, Santos NJR, Ferreira FP, Martins CM, et al. Spatial and simultaneous representative seroprevalence of anti-Toxoplasma gondii antibodies in owners and their domiciled dogs in a major city of southern Brazil. PLoS One 2017;12. doi:10.1371/journal.pone.0180906.

Brasil AW de L, Parentoni RN, Silva JG da, Santos C de SAB, Mota RA, Azevedo SS de. Risk factors and anti-Toxoplasma gondii and Neospora caninum antibody occurrence in dogs in João Pessoa, Paraíba state, Northeastern Brazil. Brazilian J Vet Parasitol 2018;27:242–7.

Caldart ET, Constantino C, Sbruzzi Pasquali AK, Benitez A do N, Hamada FN, Ferreira Dias RC, et al. Zoonosis in dogs and cats attended by the Birth Control Project: Toxoplasma gondii, Leishmania spp. and Leptospira spp., serodiagnosis and epidemiology. Semin Ciências Agrárias 2015;36:253–66. doi:10.5433/1679-0359.2015v36n1p253.

Constantino C, Pellizzaro M, Paula EFE de, Vieira TSWJ, Brandão APD, Ferreira F, et al. Serosurvey for Leishmania spp., Toxoplasma gondii, Trypanosoma cruzi and Neospora caninum in neighborhood dogs in Curitiba-Paraná, Brazil. Rev Bras Parasitol Veterinária 2016;25:504–10. doi:10.1590/s1984-29612016062.

Ferreira MU, Da Silva-Nunes M, Da Silva NS, Muniz PT, Hiramoto RM, Aureliano DP, et al. A community-based survey of human toxoplasmosis in rural Amazonia: Seroprevalence, seroconversion rate, and associated risk factors. Am J Trop Med Hyg 2009;81:171–6. doi:10.4269/ajtmh.2009.81.171.

Francisco FDM, De Souza SLP, Gennari SM, Pinheiro SR, Muradian V, Soares RM. Seroprevalence of toxoplasmosis in a low-income community in the São Paulo municipality, SP, Brazil. Rev Inst Med Trop Sao Paulo 2006;48:167–70. doi:10.1590/S0036-46652006000300009.

Furtado MM, Gennari SM, Ikuta CY, Jacomo AT de A, de Morais ZM, Pena HF de J, et al. Serosurvey of Smooth Brucella, Leptospira spp. and Toxoplasma gondii in Free-Ranging Jaguars (Panthera onca) and Domestic Animals from Brazil. PLoS One 2015;10:e0143816. doi:10.1371/journal.pone.0143816.

Magalhães FJR, Ribeiro-Andrade M, Souza FM, Lima Filho CDF, Biondo AW, Vidotto O, et al. Seroprevalence and spatial distribution of Toxoplasma gondii infection in cats, dogs, pigs and equines of the Fernando de Noronha Island, Brazil. Parasitol Int 2017;66:43–6. doi:10.1016/j.parint.2016.11.014.

Maia LP, Gómez-Hernández C, Oliveira KR de, Nomeline QSS, Aidar FL de M, Ferreira GLS. Soroprevalencia De Toxoplasmose Na Região Do Pontal Do Triangulo Mineiro, Minas Gerais, Brasil. Rev Patol Trop 2012;41:457–64. doi:10.5216/rpt.v41i4.21707.

Mareze M, do Nascimento Benitez A, Pérola Drulla Brandão A, Pinto-Ferreira F, Miura AC, Cardoso Martins FD, et al. Socioeconomic vulnerability associated to Toxoplasma gondii exposure in southern Brazil. PLoS One 2019;14:1–14. doi:10.1371/journal.pone.0212375.

Meireles LR, Galisteo AJ, Pompeu E, Andrade HF. Toxoplasma gondii spreading in an urban area evaluated by seroprevalence in free-living cats and dogs. Trop Med Int Heal 2004;9:876–81. doi:10.1111/j.1365-3156.2004.01280.x.

Minervino AHH, Cassinelli ABM, de Lima JTR, Soares HS, Malheiros AF, Marcili A, et al. Prevalence of Anti- Neospora caninum and Anti-Toxoplasma gondii Antibodies in Dogs From Two Different Indigenous Communities in the Brazilian Amazon Region . J Parasitol 2013;98:1276–8. doi:10.1645/ge-3151.1.

Passos ADC, Bollela VR, Furtado JMF, de Lucena MM, Bellissimo-Rodrigues F, Paula JS, et al. Prevalence and risk factors of toxoplasmosis among adults in a small Brazilian city. Rev Soc Bras Med Trop 2018;51:781–7. doi:10.1590/0037-8682-0214-2017.

de Paula Dreer MMK, Gonçalves D, da Silva Caetano IC, Gerônimo E, Menegas PH, Bergo D, et al. Toxoplasmosis, leptospirosis and brucellosis in stray dogs housed at the shelter in Umuarama municipality, Paraná, Brazil. J Venom Anim Toxins Incl Trop Dis 2013;19:23. doi:10.1186/1678-9199-19-23.

Paulan S de C, Lins AG de S, Tenório M da S, Silva DT da, Pena HF de J, Machado RZ, et al. Seroprevalence rates of antibodies againstLeishmania infantum and other protozoan and rickettsial parasites in dogs. Rev Bras Parasitol Veterinária 2013;22:162–6. doi:10.1590/s1984-29612013000100031.

Pinto-Ferreira F, Pasquali AKS, Thomaz-Soccol V, Mitsuka-Breganó R, Caldart ET, Leandro A de S, et al. Epidemiological relevance of dogs for the prevention of Toxoplasma gondii , Neospora caninum and Leptospira spp . Brazilian J Vet Parasitol 2019;Ahead of p:1–12.

doi:https://doi.org/10.1590/S1984-29612019043 Epidemiological.

Raimundo JM, Guimarães A, Moraes LM de B, Santos LA, Nepomuceno LL, Barbosa SM, et al. Toxoplasma gondii and Neospora caninum in dogs from the state of Tocantins: serology and associated factors. Rev Bras Parasitol Veterinária 2015;24:475–81. doi:10.1590/s1984-29612015068.

Rodrigues JY, Almeida A do BPF de, Boa Sorte E da C, Gasparetto ND, Cruz FACS da, Sousa VRF. Seroprevalence of Toxoplasma gondii in dogs of riverside communities of Mato Grosso Pantanal, Brazil. Rev Bras Parasitol Veterinária 2016;25:531–5. doi:10.1590/s1984-29612016067.

Saldanha-Elias AM, Silva MA, Silva VO, Amorim SLA, Coutinho AR, Santos HA, et al. Prevalence of Endoparasites in Urban Stray Dogs from Brazil Diagnosed with Leishmania, with Potential for Human Zoonoses. Acta Parasitol 2019. doi:10.2478/s11686-019-00043-x.

Santos TR, Costa AJ, Toniollo GH, Luvizotto MCR, Benetti AH, Santos RR, et al. Prevalence of anti-Toxoplasma gondii antibodies in dairy cattle, dogs, and humans from the Jauru micro-region, Mato Grosso state, Brazil. Vet Parasitol 2009;161:324–6. doi:10.1016/j.vetpar.2009.01.017.

Seabra NM de, Pereira VF, Kuwassaki MV, Benassi JC, Oliveira TMF de S. Toxoplasma gondii, Neospora caninum and Leishmania spp. serology and Leishmania spp. PCR in dogs from Pirassununga, SP. Rev Bras Parasitol Veterinária 2015;24:454–8. doi:10.1590/s1984-29612015046.

Silva RC da, Souza LC de, Langoni H, Tanaka EM, Lima VY de, Silva AV da. Risk factors and presence of antibodies to Toxoplasma gondii in dogs from the coast of São Paulo State, Brazil. Pesqui Veterinária Bras 2010;30:161–6. doi:10.1590/s0100-736x2010000200011.

Souza IB de, Fernandes PR, Silva TRM, Santos CVB, Silva NMM da, Ubirajara Filho CRC, et al. Seroprevalence of Neospora caninum and Toxoplasma gondii in dogs from an urban area of North-eastern Brazil: a spatial approach. Rev Soc Bras Med Trop 2019;52:3–5. doi:10.1590/0037-8682-0440-2018.

Tuon FF, Wollmann LC, Pegoraro D, Gouveia AM, Andrejow AP, Schultz AT, et al. Seroprevalence of Toxoplasma gondii, cytomegalovirus and Epstein Barr virus in 578 tissue donors in Brazil. J Infect Public Health 2019;12:289–91. doi:10.1016/j.jiph.2018.07.001.

Varandas NP, Rached PA, Henrique G, Costa N, Souza LM De, Castagnolli KC, et al. Freqüência de anticorpos anti-Neospora caninum e anti-Toxoplasma gondii em cães da região nordeste do Estado de São Paulo . Correlação com neuropatias Frequence of antibodies for Neospora caninum and Toxoplasma gondii in dogs in northest of São Paulo Stat 2001:105–11.

Vitaliano SN, De Mendonça GM, de Sandres FAM, de Camargo JSAA, de Tarso P, Basano S de A, et al. Epidemiological aspects of Toxoplasma gondii infection in riverside communities in the Southern Brazilian Amazon. Rev Soc Bras Med Trop 2015;48:301–6. doi:10.1590/0037-8682-0040-2015.
