# Supplementary material for: Spatial serosurvey of anti-Toxoplasma gondii antibodies in individuals with animal hoarding disorder and their dogs in Southern Brazil
Source: PLoS One. 2020 May 15;15(5):e0233305. doi: 10.1371/journal.pone.0233305 (PMC7228105; doi:10.1371/journal.pone.0233305)
Supplement: S2 Table — (DOCX) [file pone.0233305.s002.docx]

**S2 Table:** **Bivariate analysis of epidemiological data and seropositivity for anti-*T.* *gondii* in households and individuals with animal hoarding disorder (AHD) in Curitiba, Paraná, Brazil.**

| **Households (N=11)** | | **Positive**  **n (%)** | **Total** | **OR** | **95% CI** | **p-value** |
| --- | --- | --- | --- | --- | --- | --- |
| Presence of cats at the household | Yes | 7 (87.5) | 8 | 3.5 | 0.14-84.69 | 0.49 |
|  | No | 2 (66.7) | 3 |  |  |  |
| Vegetable garden | Yes | 4 (100.0) | 4 | - | - | * |
|  | No | 5 (71.4) | 7 |  |  |  |
| Open sewer near the house | Yes | 4 (80.0) | 5 | 0.8 | 0.03-17.19 | 0.72 |
|  | No | 5 (83.3) | 6 |  |  |  |
| Cat hoarding | Yes | 3 (75.0) | 4 | 0.5 | 0.02-11.08 | 0.61 |
|  | No | 6 (85.7) | 7 |  |  |  |
| Object hoarding | Yes | 2 (100.0) | 2 | - | - | * |
|  | No | 7 (77.8) | 9 |  |  |  |
| Presence of feces on floor | Yes | 4 (100.0) | 4 | - | - | * |
|  | No | 5 (71.4) | 7 |  |  |  |
| Food preparation place | Clean | 4 (80.0) | 5 | - | - | * |
|  | Dirty | 1 (100.0) | 1 |  |  |  |
| Remains of food | Yes | 3 (100.0) | 3 | - | - | * |
|  | No | 6 (75.0) | 8 |  |  |  |
| House features | Brick | 6 (85.7) | 7 |  |  | Ref |
|  | Wood | 2 (66.7) | 3 | 0.3 | 0.01-8.18 | 0.53 |
|  | Mixed | 1 (100.0) | 1 | - | - | * |
| Backyard features | Cemented | 3 (60.0) | 5 |  |  | Ref |
|  | Mixed | 4 (100.0) | 4 | - | - | * |
|  | Soil and grass | 2 (100.0) | 2 | - | - | * |
| Trash in the yard | Yes | 4 (80.0) | 5 | 0.8 | 0.03-17.19 | 0.72 |
|  | No | 5 (83.3) | 6 |  |  |  |
| **Individuals with AHD (N=19)** | | **Positive**  **n (%)** | **Total** | **OR** | **95% CI** | **p-value** |
| Gloves to collect the animal feces | Yes | 0 (0.0) | 1 | - | - | * |
|  | No | 7 (43.8) | 16 |  |  |  |
| Habit of eating raw or undercooked meat | Yes | 1 (50.0) | 2 | 2.2 | 0.11-42.73 | 0.56 |
|  | No | 5 (31.2) | 16 |  |  |  |
| Knowledge about toxoplasmosis | Yes | 4 (40.0) | 10 | 2.0 | 0.26-15.38 | 0.63 |
|  | No | 2 (25.0) | 8 |  |  |  |
| Cat hoarding | Yes | 1 (20.0) | 5 | 0.3 | 0.02-3.80 | 0.60 |
|  | No | 6 (42.9) | 14 |  |  |  |
| Object hoarding | Yes | 1 (100.0) | 1 | - | - | * |
|  | No | 6 (33.3) | 18 |  |  |  |
| Presence of cats in the household | Yes | 6 (40.0) | 15 | 2.0 | 0.16-24.06 | 0.52 |
|  | No | 1 (25.0) | 4 |  |  |  |

* There was no sufficient exposed and no exposed to proceed the analysis.
