# Supplementary material for: Spatial serosurvey of anti-Toxoplasma gondii antibodies in individuals with animal hoarding disorder and their dogs in Southern Brazil
Source: PLoS One. 2020 May 15;15(5):e0233305. doi: 10.1371/journal.pone.0233305 (PMC7228105; doi:10.1371/journal.pone.0233305)
Supplement: S1 Questionnaire — (PDF) [file pone.0233305.s003.pdf]

S1 Questionnaire: Epidemiological questionnaire used to investigation of seroprevalence of anti-*T. gondii* antibodies and associated factors in individuals with animal hoarding disorder and their dogs in Curitiba.

(Translated to English)

Researcher: ____ Date: ___/___/_____
Case identification: _________________ Dog identification: ______________________
Address: ____

HOUSEHOLDS

1. Presence of cats in the household: (   ) Yes (   ) No
2. Presence of vegetable garden: (   ) Yes (   ) No
3. Presence of open sewer or stream near the house: (   ) Yes (   ) No
4. Cat hoarding: (   ) Yes (   ) No
5. Object hoarding: (   ) Yes (   ) No
6. Is there feces on floor: (   ) Yes (   ) No
7. Is there remains of food around the house: (   ) Yes (   ) No
8. Is there trash in the yard: (   ) Yes (   ) No
9. Situation of food preparation place (clean or dirty),
10. House features (brick, wood, or mixed), and
11. Backyard features (cemented, soil, grass, or mixed) were investigated.

INDIVIDUALS WITH ANIMAL HOARDING DISORDER

12. Do you use of gloves to collect the animals' feces? (   ) Yes (   ) No
13. Do you have the habit of eating raw or undercooked meat? (   ) Yes (   ) No
14. Previously reference of hearing about toxoplasmosis: (   ) Yes (   ) No

DOGS

15. Dog's living place: (   ) Inside the home (   )Backyard (   ) Both
16. Food type provided to dogs: (   ) Commercial dog food (   ) Homemade food and commercial dog food
17. Dog's feeding place: (   ) Food bowls (   ) Directly on the floor

(Original language)

Pesquisador: ____ Data: ___/___/_____
Identificação do caso: ________________ Identificação do cão: __________________
Endereço: ____

DOMICÍLIOS

1. Presença de gatos no local: ( ) Sim ( ) Não
2. Presença de horta no quintal: ( ) Sim ( ) Não
3. Presença de córrego ou esgoto aberto próximo da casa: ( ) Sim ( ) Não
4. Acumulação de gatos: ( ) Sim ( ) Não
5. Acumulação de objetos: ( ) Sim ( ) Não
6. Há fezes no chão: ( ) Sim ( ) Não
7. Há restos de comida espalhados pela casa: ( ) Sim ( ) Não
8. Há lixo espalhado pelo quintal: ( ) Sim ( ) Não
9. Situação do local de preparação dos alimentos: ( ) Limpo ( ) Sujo
10. Característica da casa: ( ) Alvenaria ( ) Madeira ( ) Mista
11. Característica do quintal: ( ) Cimentado ( ) Terra e grama ( ) Misto

INDIVÍDUOS COM ACUMULAÇÃO DE ANIMAIS

12. Usa luvas para recolher as fezes dos animais? ( ) Sim ( ) Não
13. Tem o hábito de consumir carne crua ou mal passada? ( ) Sim ( ) Não
14. Já ouviu falar em toxoplasmose? ( ) Sim ( ) Não

CÃES

15. Local que o animal vive: ( ) Dentro de casa ( ) Quintal ( ) Ambos
16. Tipo de alimento fornecido aos animais: ( ) Ração ( ) Comida e ração
17. Local de alimentação dos animais: ( ) Potes próprios ( ) No chão
